# Metabolomics of sorghum roots during nitrogen stress reveals compromised metabolic capacity for salicylic acid biosynthesis

**DOI:** 10.1002/pld3.122

**Published:** 2019-03-14

**Authors:** Amy M. Sheflin, Dawn Chiniquy, Chaohui Yuan, Emily Goren, Indrajit Kumar, Max Braud, Thomas Brutnell, Andrea L. Eveland, Susannah Tringe, Peng Liu, Stephen Kresovich, Ellen L. Marsh, Daniel P. Schachtman, Jessica E. Prenni

**Affiliations:** ^1^ Department of Horticulture and Landscape Architecture Colorado State University Fort Collins Colorado; ^2^ Joint Genome Institute Department of Energy Walnut Creek California; ^3^ Bioinformatics & Computational Biology Iowa State University Ames Iowa; ^4^ Donald Danforth Plant Science Center St. Louis Missouri; ^5^ Plant and Environmental Genetics and Biochemistry Departments Clemson University Clemson South Carolina; ^6^ Center for Biotechnology University of Nebraska‐Lincoln Lincoln Nebraska

**Keywords:** metabolism, metabolomics, microbiome, nitrogen, rhizosphere, roots, salicylic acid, sorghum, stress

## Abstract

Sorghum (*Sorghum bicolor* [L.] Moench) is the fifth most productive cereal crop worldwide with some hybrids having high biomass yield traits making it promising for sustainable, economical biofuel production. To maximize biofuel feedstock yields, a more complete understanding of metabolic responses to low nitrogen (N) will be useful for incorporation in crop improvement efforts. In this study, 10 diverse sorghum entries (including inbreds and hybrids) were field‐grown under low and full N conditions and roots were sampled at two time points for metabolomics and 16S amplicon sequencing. Roots of plants grown under low N showed altered metabolic profiles at both sampling dates including metabolites important in N storage and synthesis of aromatic amino acids. Complementary investigation of the rhizosphere microbiome revealed dominance by a single operational taxonomic unit (OTU) in an early sampling that was taxonomically assigned to the genus *Pseudomonas*. Abundance of this *Pseudomonas* OTU was significantly greater under low N in July and was decreased dramatically in September. Correlation of *Pseudomonas* abundance with root metabolites revealed a strong negative association with the defense hormone salicylic acid (SA) under full N but not under low N, suggesting reduced defense response. Roots from plants with N stress also contained reduced phenylalanine, a precursor for SA, providing further evidence for compromised metabolic capacity for defense response under low N conditions. Our findings suggest that interactions between biotic and abiotic stresses may affect metabolic capacity for plant defense and need to be concurrently prioritized as breeding programs become established for biofuels production on marginal soils.

## 1 INTRODUCTION

Sorghum (*Sorghum bicolor* [L.] Moench) is the fifth most productive cereal crop worldwide with a variety of uses including animal forage, sugar production, and more recently, lignocellulosic biomass for bioenergy (Rooney, Blumenthal, Bean, & Mullet, 2007). Sorghum's high biomass yield and drought tolerance potential make it a promising feedstock for sustainable, economical biofuel production. Production strategies that utilize agricultural lands not suitable for food production and improve economic viability are a top priority for biofuel feedstocks (Langholtz, Stokes, & Eaton, 2016). Improving biomass production will require maximizing yield while reducing costly inputs, including nitrogen (N), and water (Fazio & Monti, 2011, National Research Council, 2012). Fortunately, sorghum possesses significant genetic diversity for N use efficiency (Gelli et al., 2014, 2016), yield potential and drought tolerance (Mace et al., 2013). Furthermore, the availability of a reference genome (Paterson et al., 2009), an expression atlas (Shakoor et al., 2014), and over 400,000 markers (Morris et al., 2013) identified for marker‐assisted breeding makes sorghum well‐suited for significant crop improvement.

Modern “omics” techniques function as valuable molecular discovery tools for assessing plant stress responses on a molecular level (Urano, Kurihara, Seki, & Shinozaki, 2010). The “metabolome” is described by surveying all low molecular weight metabolites generated as a result of gene‐regulated and environmentally induced responses (Brunetti, George, Tattini, Field, & Davey, 2013; Ganie et al., 2015). Since metabolites are one measurable endpoint of all cellular regulatory activities, they have been described as “the ultimate response of biological systems to genetic or environmental changes” (Fiehn, 2002). Thus, metabolomics is a robust approach for determining the molecular phenotype, especially in assessing response during both abiotic (Ganie et al., 2015; Sánchez‐Martín et al., 2015) and biotic (Scandiani et al., 2014) stress and in identifying potential targets for metabolic engineering (Tsogtbaatar, Cocuron, Sonera, & Alonso, 2015). For example, previous research has associated biomass accumulation with specific sorghum leaf metabolites that vary across different sorghum genetic lines (Turner et al., 2016). However, relationships between root metabolites and biomass yields in field‐grown sorghum have been largely ignored.

As a synergistic approach to plant breeding, beneficial microbes offer an underutilized opportunity to improve plant performance, especially on marginal lands with minimal inputs (Coleman‐Derr & Tringe, 2014). Plant growth and health may be improved through interactions with beneficial soil microorganisms via a number of mechanisms including increased nutrient availability, production of protective or growth‐promoting enzymes and compounds, and competitive exclusion of pathogens (Chaparro, Sheflin, Manter, & Vivanco, 2012; Farrar, Bryant, & Cope‐Selby, 2014). Under marginal soil conditions microbes may play an important role in providing nitrogen to cereals and have been shown to potentially assist sorghum with the acquisition of N in nutrient depleted environments (Carvalho, Balsemão‐Pires, Saraiva, Ferreira, & Hemerly, 2014; Kochar & Singh, 2016; Rupaedah, Anas, Santosa, Sumaryono, & Budi, 2016). Next‐generation sequencing provides an opportunity to identify potential microbes that may provide these benefits, even those not amenable to rapid laboratory cultivation (Knief, 2014). Sorghum‐associated bacterial communities have been recently reviewed (Kochar & Singh, 2016) and one study identified N application as a primary factor in determining N‐fixing community structure in the sorghum rhizosphere (Rodrigues Coelho et al., 2007). Conversely, detrimental microorganisms present additional challenges to maximizing yield in marginal environments that lack the optimal amounts of water or nitrogen (van der Heijden, Bardgett, & van Straalen, 2008). A better understanding of these interactions among soil nutrients, bacteria, and sorghum yields will be an important part of the development of sustainable and economical biofuel production on marginal land.

The primary goal of this study is to improve our understanding of the root metabolic response to low N in field grown sorghum. Utilizing an integrative approach, we incorporated data from agronomic traits, the root metabolome and the rhizosphere microbiome of 10 sorghum lines (including inbreds and hybrids) grown under conditions of both low and full N and sampled on two dates. Taken together, our findings highlight an array of metabolic disadvantages due to N stress that may reduce capacity for plant defense. Therefore, secondary biotic stresses should be considered as a potential consequence of abiotic stresses, such as low N availability.

## 2 EXPERIMENTAL PROCEDURES

### 2.1 Field description and experimental design

This study was conducted at Eastern Nebraska Research and Extension Center (ENREC) located near Mead, Nebraska in the United States during the summer of 2015. The low N field (GPS coordinates 41.163166, −96.424108) had not had N applied for more than 20 years and was in an oat/sorghum rotation with oat forage removed each year. The full N field (41.156414, −96.408031) had eighty pounds per acre of N in the form of anhydrous ammonia applied early in the spring and was in a soybean/sorghum rotation. Both fields were planted on June 2, 2015.

### 2.2 Plant germplasm selection

Ten diverse entries of sorghum, representing a range of materials based on racial, geographic, and breeding status criteria, were selected for testing (Table 1). All lines represent bioenergy types and likely would be candidates for biofuels production. Entries are identified by plant introduction numbers of the U.S. National Plant Germplasm System or hybrid numbers from Clemson University (for more information or germplasm, please contact Stephen Kresovich at Clemson).

**Table 1:** Sorghum accessions utilized for this study

Genotype	Type	Race	Source	Breeding status
PI 297130	Energy	Caudatum	Uganda	Inbred
PI 505735	Energy	Caudatum	Zambia	Inbred
PI 506030	Energy	Guinea	Togo	Inbred
PI 510757	Energy	Durra	Cameroon	Inbred
PI 642998	Sweet	Bicolor	U.S.	Inbred
PI 655972	Energy	Kafir	U.S. (Kansas)	Inbred
C126	Energy	Guinea	U.S. (South Carolina)	Hybrid
C225	Energy	Caudatum	U.S. (South Carolina)	Hybrid
CO53	Energy	Guinea	U.S. (South Carolina)	Hybrid
CO56	Energy	Guinea	U.S. (South Carolina)	Hybrid

### 2.3 Field sample collection

Soil, rhizosphere, and root samples were collected from each field two times during the growing season. The sampling dates were July 22 and September 15, 2015. Two plants per genotype were excavated from the top 30 cm of soil using a shovel at two different locations in each plot as described previously (McPherson, Wang, Marsh, Mitchell, & Schachtman, 2018). Soil was removed from the roots using a tiller and collected. A variety of roots including crown, seminal, and primary roots were excised from two plants and placed in a 50 ml Falcon tube containing 35 ml of phosphate buffer (6.33 g/L NaH_2_PO_4_, 8.5 g/L Na_2_HPO_4_ anhydrous, 200 μl/L Silwet L‐77). Roots were then shaken for 1–2 min to remove some of the rhizosphere soil. Roots were then separated for downstream analyses: DNA extraction and microbiome analysis, and metabolite analysis. Rhizosphere soil and roots for DNA extraction were stored in 50 ml Falcon tubes on ice for transport to the laboratory.

Samples were collected for biomass analysis on October 8, 2015. An above ground portion of plants from 1 square meter for each plot was weighed for fresh biomass measurements. The same samples were oven dried in paper sacks until reaching a stable water content before weighing for dry biomass measurements.

### 2.4 Laboratory preparation of roots, soil, and rhizosphere

Roots that were brought back to the laboratory on regular ice were surface sterilized by rinsing for 30 s in 5.25% sodium hypochlorite + 0.01% Tween 20, followed by a 30‐s rinse in 70% ethanol, followed by three rinses in sterile ultrapure water. Roots were blotted dry on a clean paper towel, placed in a 15 ml tube, frozen at −80°C, and then ground in liquid N prior to DNA extraction.

The rhizosphere samples were filtered through a sterile 100 μm mesh filter (Fisher Scientific, USA), into a clean 50 ml tube. The rhizosphere was pelleted at 3,000 × *g* for 10 min at room temperature. The pellet was resuspended in 1.5 ml phosphate buffer (6.33 g/L NaH_2_PO_4_, 8.5 g/L Na_2_HPO_4_ anhydrous), and transferred to a sterile 2 ml microfuge tube. The rhizosphere was re‐pelleted by spinning tubes for 5 min at full speed. The supernatant was drained off and the rhizosphere soil pellet was stored at −20°C until DNA extraction.

A small 2 ml tube of soil was removed carefully to avoid any root pieces and stored for DNA extraction at −20°C. Soil was then sieved through US Standard Sieve #4, 4750 micron, followed by Sieve #8, 2360 micron to remove debris and roots. Approximately 100 g of sieved soil was sent for soil analysis (Ward Labs, Kearney, NE) to determine the organic carbon and nutrient concentrations of the soil.

### 2.5 DNA extraction of soil, rhizosphere, and root samples

DNA was extracted from soil and rhizosphere samples using PowerSoil‐htp 96 Well Soil DNA Isolation Kit (MoBio, Carlsbad, CA, USA) following the manufacturer's protocol. DNA was extracted from roots with PowerPlant Pro‐htp 96 Well DNA Isolation Kit (MoBio, Carlsbad, CA, USA) following the manufacturer's protocol. The DNA was quantified with the Quantifluor dsDNA reagent (Promega) following the manufacturer's protocol.

### 2.6 Amplification and Illumina sequencing of 16S tag sequences

DNA was quantified and amplified in 96 well plates with single indexed primers targeting the V4 region of the bacterial 16S rRNA gene (Walters et al., 2015). Chloroplast and mitochondrial peptide nucleic acid (PNA) blockers were used to prevent chloroplast and mitochondrial amplification in all samples (Lundberg, Yourstone, Mieczkowski, Jones, & Dangl, 2013). Amplified samples were multiplexed at 184 samples per PE 2 × 300 Illumina MiSeq sequencing run. Data from the sequencer was demultiplexed and processed through bbduk for end trimming, quality filtering, and masking (https://jgi.doe.gov/data-and-tools/bbtools/bb-tools-user-guide/bbduk-guide/). High quality reads were processed by iTagger version 2.2 (Tremblay et al., 2015). Version 2.2 processes sequencing amplicon data by iterative clustering of reads at 99%, 98%, and 97% identity using the USEARCH software suite, and then performing taxonomic assignment of each OTU (Edgar, 2010). The source code for iTagger is available on Bitbucket: http://bitbucket.org/berkeleylab/jgi_itagger. For normalization purposes and to remove low abundance OTUs, we kept OTUs in the downstream analysis that had at least two reads in at least five samples and normalized the remaining dataset by randomly subsampling each sample to a consistent read depth of 20,000 reads per sample. The normalized OTU table was analyzed downstream using MicrobiomeAnalyst (http://www.microbiomeanalyst.ca/) (Dhariwal et al., 2017) to produce PCoA visualizations of the data. Statistical analyses of the normalized OTU table, including PERMANOVA and ANCOM (Mandal et al., 2015) were performed in QIIME2 (https://qiime2.org) (Bolyen et al., 2018).

### 2.7 Sample preparation and extraction for metabolomics analysis

Immediately upon arrival, sorghum tissue samples were stored at −80°C with subsequent lyophilization for further analysis. Lyophilized sorghum tissue was homogenized in 5 ml polypropylene tubes using stainless steel beads and the Bullet Blender^®^ Storm5 homogenizer (Next Advance, Averill Park, NY). A 19–21 mg portion of the lyophilized and homogenized tissue was weighed into a 2 ml glass vial. Extraction of this tissue is visually summarized in Supporting Information Figure S5. Phytohormones were extracted by adding 1 ml of methyl‐tert‐butyl‐ether (MTBE) solution containing 6:3:1 MTBE:methanol:water (v/v/v) and vortexing at 4°C for 60 min. Next, glass vials were centrifuged for 15 min at 3,500 rpm. A 400 μl aliquot of the extraction was dried completely under N gas, resuspended in 100 μl methanol and stored in a glass insert in a 2 ml glass vial at −80°C until further analysis. To separate organic and aqueous layers, 350 μl of water was added to the remaining extract and vortexed for 30 min at 4°C. Glass vials were centrifuged at 2,750 rpm at 4°C for 15 min and the organic layer was transferred into a separate 2 ml glass vial. The aqueous layer was transferred to an Ambion^®^ filter cartridge (Thermo Fisher Scientific, USA) and centrifuged briefly to pass the aqueous extract through the column. The filtrate was transferred to a separate glass vial and stored at −80°C until further analysis. In addition, a pooled extract was created by combining equal volumes of each sample into one glass vial for use as a consistent representative quality control sample (QC).

### 2.8 Targeted UPLC‐MS/MS phytohormone analysis

Two microliters of monophasic plant extract were injected in an ACQUITY UPLC System, equipped with an ACQUITY Binary Solvent Manager (20 μl sample loop, partial loop injection mode; Waters Corporation). An Acquity UPLC T3 column (1 × 100 mm, 1.8 μM; Waters Corporation) was used for chromatographic separation. Mobile phase A consisted of LC‐MS grade water with 0.1% formic acid and mobile phase B was 100% acetonitrile. The elution gradient was initially 0.1% B for 1 min, which was increased to 55.0% B at 12 min and further increased to 97.0% B at 15 min, then decreased to 0.1% B at 15.5 min. The column was re‐equilibrated for 4.5 min for a total run time of 20 min. The flow rate was set to 120 μl/min and the column temperature was maintained at 45°C. Mass spectrometry was performed on a Xevo TQ‐S triple quadrupole MS (Waters Corporation) operated in selected reaction monitoring (SRM) mode (Supporting Information Table S5).

Skyline bioinformatics software (*MacLean, Bioinformatics* 2010) was used to detect and integrate peak areas and to calculate linear regression of analytical standards used for quantification. Prior to quantification, each analyte peak area was normalized to the internal standard (Supporting Information Table S6).

All raw data files were imported into the Skyline open source software package (MacLean et al., 2010). Each target analyte was visually inspected for retention time and peak area integration. Peak areas were exported to Excel and absolute quantitation was determined by using the linear regression equation generated for each compound from the calibration curve. To make the calibration curve, analytical standards were diluted in pure methanol serially from 400 ng/ml to 0.54 ng/ml before adding an equal amount of every internal standard to each vial. The linear regression equation of the analytical standard curve was used to convert the normalized peak area to quantity (ng/ml) for each analyte. The values were then adjusted for precise weight of root tissue for each sample and reported as ng/g root tissue.

### 2.9 Non‐targeted reverse phase UPLC‐MS/MS analysis

A 200 μl aliquot of the organic layer was dried and resuspended in 100 μl of methanol and toluene (1:4, v/v). Single injections of 3 μl of extract were made on an Acquity UPLC system (Waters Corporation) in discrete, randomized blocks. The pooled QC was injected after every 10 sample injections.

Separation was performed with an Acquity UPLC CSH Phenyl Hexyl column (1.7 μM, 1.0 × 100 mm; Waters Corporation), using a gradient from solvent A (water, 0.1% formic acid) to solvent B (acetonitrile, 0.1% formic acid). Injections were made in 100% A, held at 100% A for 1 min, ramped to 98% B over 12 min, held at 98% B for 3 min, and then returned to starting conditions over 0.05 min and allowed to re‐equilibrate for 3.95 min, with a 200 μl/min constant flow rate. The column and samples were held at 65°C and 6°C, respectively. The column eluent was infused into a Xevo G2 Q‐TOF‐MS (Waters Corporation) with an electrospray source in positive mode, scanning 50–2,000 *m*/*z* at 0.2 s per scan, alternating between MS (6 V collision energy) and MSE mode (15–30 V ramp). Calibration was performed using sodium iodide with 1 ppm mass accuracy. The capillary voltage was held at 2,200 V, source temp at 150°C, and N desolvation temp at 350°C with a flow rate of 800 L/hr.

### 2.10 Non‐targeted UPLC‐MS/MS HILIC analysis

Single injections of 3 μl of the aqueous extract were made on a Waters Acquity UPLC system in discrete, randomized blocks. The pooled QC was injected every after every 10 injections. Separation was performed using a ZIC‐pHilic (5 μM, 2.0 × 150 mm; EMD Millipore), using a gradient from solvent A (acetonitrile) to solvent B (water, 10 mM Ammomium Bicarbonate, pH 9.6). Flow rate was 0.27 ml/min unless noted otherwise, and the column was held at 50°C. The gradient is as follows: time (*t*) = 0 min, 10% A; *t* = 1.5 min, 10% A; *t* = 8.5 min, 38% A; *t* = 11 min, 60% A; *t* = 11.5 min, 100% A, 0.2 ml/min flow; *t* = 16.5 min, 100% A; *t* = 17 min, 10% A; *t* = 18 min, 10% A, 0.6 ml/min flow; *t* = 22 min 10% A; *t* = 22.5 min, 10% A, 0.27 ml/min flow; *t* = 23 min, 10% A, end of equilibration. The column eluent was infused into a Xevo G2 Q‐TOF‐MS (Waters Corporation) with an electrospray source in negative ionization mode, scanning 50–1,200 *m*/*z* at 0.2 s per scan, alternating between MS (6 V collision energy) and MSE mode (15–30 V ramp). Calibration was performed using sodium formate with 1 ppm mass accuracy. The capillary voltage was held at 2,200 V, source temperature at 150°C, and N desolvation temperature at 350°C with a flow rate of 800 L/hr.

### 2.11 Non‐targeted GC‐MS analysis

A 200 μl aliquot of the aqueous layer was dried down completely under N_2_ (g). The dried samples were resuspended in 50 μl of pyridine containing 25 mg/ml of methoxyamine hydrochloride (Sigma), incubated at 60°C for 45 min, vigorously vortexed for 30 s, sonicated for 10 min, and incubated for an additional 45 min at 60°C. Next, samples were cooled to room temperature and briefly centrifuged. Then, 50 μl of *N*‐methyl‐*N*‐trimethylsilyltrifluoroacetamide with 1% trimethylchlorosilane (MSTFA + 1% TMCS, Thermo Fisher Scientific) was added and the samples were vigorously vortexed for 30 s and then incubated at 60°C for 30 min. Metabolites were separated and detected using a Trace 1310 GC coupled to an ISQ mass spectrometer (Thermo Fisher Scientific). Samples (1 μl) were injected at a 10:1 split ratio onto a 30 m TG‐5MS column (0.25 mm i.d., 0.25 μm film thickness; Thermo Fisher Scientific) with a 1.2 ml/min helium gas flow rate. The gas chromatography inlet was held at 285°C. The oven program started at 80°C for 30 s, followed by a ramp of 15°C/min to 330°C, and an 8‐min hold. Masses between 50–650 *m*/*z* were scanned at 5 scans/s under electron impact ionization. Transfer line and ion source were held at 300 and 260°C, respectively.

### 2.12 Metabolomics data analysis

GC‐MS and LC‐MS data sets were processed independently using the R statistics software (R Core Team, 2013) as described previously (Yao, Sheflin, Broeckling, & Prenni, 2019). Briefly, processing steps follow: (a) XCMS software defined a matrix of molecular features (Smith, Want, O'Maille, Abagyan, & Siuzdak, 2006), (b) samples were normalized to total ion current, (c) RAMClust package for R clustered covarying and co‐eluting features into spectra (Broeckling, Afsar, Neumann, Ben‐Hur, & Prenni, 2014), (d) RAMSearch software (Broeckling et al., 2016) allowed annotation by searching spectra against internal and external databases. Databases used for annotations included golm (http://gmd.mpimp-golm.mpg.de/), NISTv14 (http://www.nist.gov), and MassBank (http://www.massbank.jp). Skyline software (MacLean et al., 2010) was used for peak picking, integration, normalization, and quantification of phytohormones. Principal component analysis (PCA) was performed using only annotated metabolites using mean‐centered and pareto‐scaled data in SIMCA v14 (Umetrics, Umea, Sweden). The biplot from the PCA analysis was also created in SIMCA using correlation scaling so that scores and loadings could be presented together to display metabolites driving variation between N treatment groups. The list of scores and loadings coordinates was then plotted in Prism 7 (GraphPad, La Jolla, California, US). Prism was used to size loading metabolites according to loading value and only values greater than 0.5 on either coordinate axis were labeled with a metabolite name. The pathway analysis tool in MetaboAnalyst (http://www.metaboanalyst.ca/) was used to identify important KEGG pathways modeling metabolic effects under low and full N conditions (Xia, Sinelnikov, Han, & Wishart, 2015). The tool was used to prioritize KEGG reference pathways of interest rather than to determine significant metabolic changes due to treatment, so cutoff was set at *p*‐value < 0.05 (ignoring adjustment for multiple testing).

### 2.13 RNA extraction, RNA‐seq library preparation, and data analysis

Frozen root samples were ground in liquid nitrogen with mortar and pestle. Equal parts of extraction buffer and 1:1 acidic phenol:chloroform were added and vortexed at 4°C for 1 hr. Samples were then centrifuged for 30 min and the aqueous layer was transferred to an RNase‐free tube, combined with equal volume of 24:1 Chloroform:Isoamyl alcohol, and vortexed. Samples were centrifuged for 15 min, aqueous layer transferred to a RNase‐free tube and mixed well with 1/3 volume of 8M LiCl. Samples were incubated overnight at 4°C and centrifuged for 30 min. Supernatent was discarded and the pellet was washed twice with ice cold 80% ethanol, mixing and centrifuging for 15 min following each wash. RNA was resuspended in 30 μl of nuclease‐free water. Samples were DNase treated using the Turbo DNase kit (Ambion) and quantified by Nanodrop. A subset of samples were quality checked using the Agilent 2100 Bioanalyzer RNA 6000 nano kit.

RNA‐seq libraries were prepared using the NEBNext Ultra Directional RNA Library Prep Kit for Illumina (New England Biolabs). Libraries were quantified using Qubit dsDNA HS Assay kit (Thermo Fischer) and quality checked using the Agilent 2100 Bioanalyzer DNA HS chip. All libraries were indexed and pooled to equal concentrations and 100 bp single‐end reads sequenced on two lanes of the Illumina HiSeq4000 at the University of Illinois Keck Center.

### 2.14 Statistical analysis

Differences between N treatments for agronomic traits were tested using a two‐tailed, unpaired Mann–Whitney (nonparametric) test (Mann & Whitney, 1947). For metabolite data, the Shapiro–Wilk test was first carried out to test whether the assumption of a normal distribution is appropriate or not for log‐transformed data using shapiro test function in R. If normality was not rejected at 5% significance level, a linear mixed model was carried out to assess the effect of collection date, N level, and genotype on metabolites using the lme4 package for R statistics software (Bates, Mächler, Bolker, & Walker, 2014). Genotype was modeled as a random effect due to the small number of replicates (at most, two per genotype) while collection date, N, and their interaction were modeled as fixed effects. This approach accounts for variation attributable to genotypes within a breeding status while preserving enough degrees of freedom to perform tests on all fixed effects, and can be justified by considering the genotypes included in the study to be a sample from a range of sorghum genotypes of interest. Due to the split‐plot design of the experiment, an additional random effect for the whole‐plot error was initially included in the model but may have been excluded from the final model for some metabolites if the estimate of the variance component corresponding to whole‐plot error was zero. Based on residual plots, there was no indication of any model assumption violations. Estimates and 95% confidence intervals for mean log metabolite for each collection date and N combination and the marginal mean for N (averaged over the two collection dates) were computed with the emmeans package for R (Lenth, 2018) using the Kenward–Roger approximation. If the normality assumption was violated, a nonparametric factorial ANOVA approach was performed using art function in ARTool R package (Wobbrock, Findlater, Gergle, & Higgins, 2011). The metabolite data without log‐transformation was modeled as a function of N treatment, collection date and interaction between N treatment and collection date as fixed effects, and genotype type and biological replicates as random effects.

Multivariate analysis for date and N treatment factors was completed for both rhizosphere bacterial composition and root metabolite profiles using the adonis2 function in the vegan package (Oksanen et al., 2013) for R statistics software (R) (R Core Team, 2013). Pairwise distances for these comparisons were calculated using the Bray–Curtis ordination method (Beals, 1984). The adonis2 function has been described as a “permutational MANOVA” (Anderson, 2001; McArdle & Anderson, 2001) and offers an alternative to parametric MANOVA. A two‐way ANOVA univariate test was used to determine differential relative abundance of a single OTU in rhizosphere soil by date, treatment, or the interaction in Prism 7 (GraphPad, La Jolla, California, US). Correlation analyses utilized the cor function in R and with the nonparametric Spearman's rank option. The corrplot package (Wei et al., 2017) for R was used to visualize correlation analyses as heatmaps.

## 3 RESULTS

The aboveground biomass and heights (Table 2 and Supporting Information Data S1) were significantly reduced in the low N treatment group. The concentration of N in each field was determined via soil composition analysis (Table 3) and determined to be 7.8 ± 0.7 ppm in the low N field and 8.5 ± 0.5 ppm in the full N field. Total aboveground dry biomass (kg/ha) was reduced by 68% and total aboveground fresh biomass was reduced by 63% (kg/ha) under low N conditions. These data show that the N treatment had a strong effect on sorghum growth and that plants were experiencing N stress. The number of plants per square meter did not significantly vary by N treatment. Since crop hybridization is known to result in superior vigor and yield (Packer & Rooney, 2014; Quinby, 1963), fold change for agronomic traits in hybrids grown with low N was compared to inbreds and was calculated (Supporting Information Figure S1). This comparison did not reveal any significant differences in biomass accumulation for hybrids versus inbreds grown under low N (*p* = 0.05), therefore hybrid status was not included as a factor for other analyses.

**Table 2:** Agronomic sorghum traits related to biomass production (means, standard error of the mean, and results of Mann–Whitney by trait) *p*‐value refers to the difference between full and low N (*n* = 20)

Trait	Full nitrogen (N)	Low nitrogen (N)	*p*‐Value
Total aboveground dry biomass (kg/ha)	25,714 ± 1,491	8,346 ± 628	<0.0001
Total aboveground fresh biomass (kg/ha)	90,727 ± 6,982	33,687 ± 2,946	<0.0001
Plant height (cm)	337.6 ± 9.612	235 ± 9.278	<0.0001
Number of plants per square meter	18.49 ± 0.7608	18.89 ± 0.5414	0.461

**Table 3:** Mean soil nutrient concentrations ± standard deviation of the mean

Treatment	K (ppm)	S (ppm)	Ca (ppm)	Mg (ppm)	NO_3_ (ppm)	NH_4_ (ppm)	P (ppm)
High N	358 ± 34	13.6 ± 0.6	1,751 ± 60	252 ± 12	8.5 ± 0.5	5.0 ± 1.1	18.4 ± 3.0
Low N	251 ± 24	13.6 ± 0.9	1,489 ± 91	297 ± 44	7.8 ± 0.7	4.9 ± 0.4	23.3 ± 3.8

### 3.1 Significant metabolite variation in low N roots

**Figure 1:**
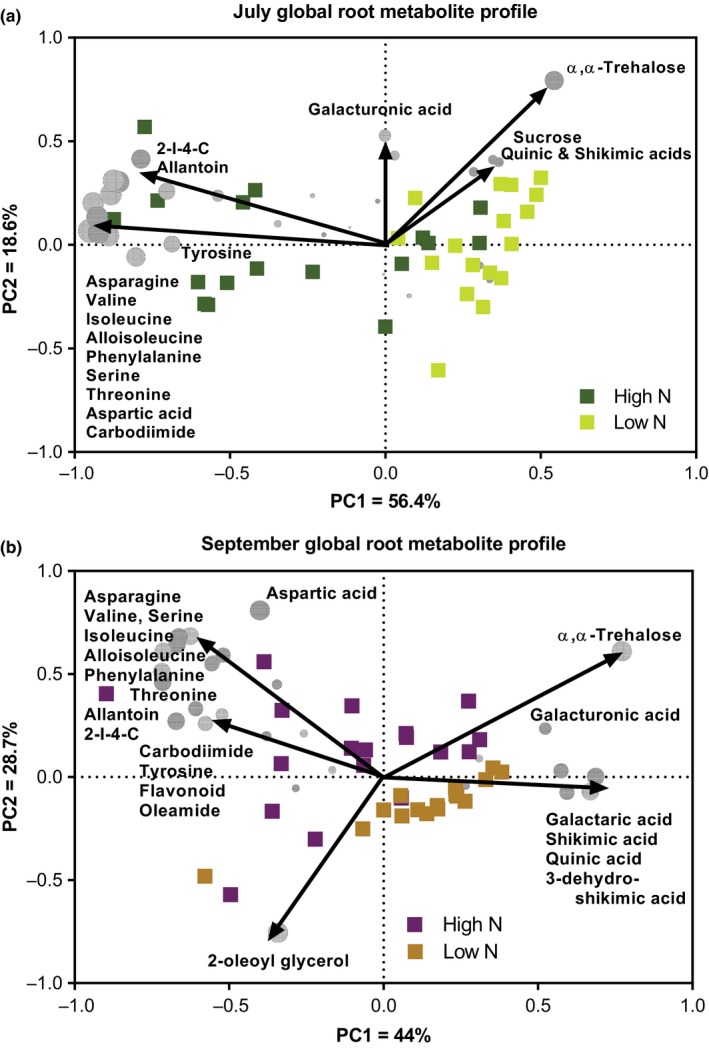
PCA biplots include scores (squares) and metabolite loadings (gray circles) of the root metabolomics analysis in (a) July and (b) September. Data from GC‐ and LC‐ MS analyses were combined. Arrows indicate the direction of influence for a specific metabolite on N treatment group separation. Circles representing metabolites are sized according to loading scores determined by the PCA analysis

A nontargeted metabolomics approach was used to evaluate biochemical variation in roots receiving low N compared to plants grown in the full N field. PCA analysis was used to visualize this variation and shows that higher trehalose, quinic, and shikimic acids drove the variation in metabolite profiles under low N conditions (Figure 1). Roots from the full N field were higher in asparagine, other amino acids, allantoin, 2‐ imidazolidone ‐4‐carboxylic acid (2‐I‐4‐C), and carbodiimide in both July and September. Permutational MANOVA (PERMANOVA) analysis revealed that the global metabolite profile was significantly different between low versus full N in both July and September (*p* < 0.01; Figure 1a,b).

**Figure 2:**
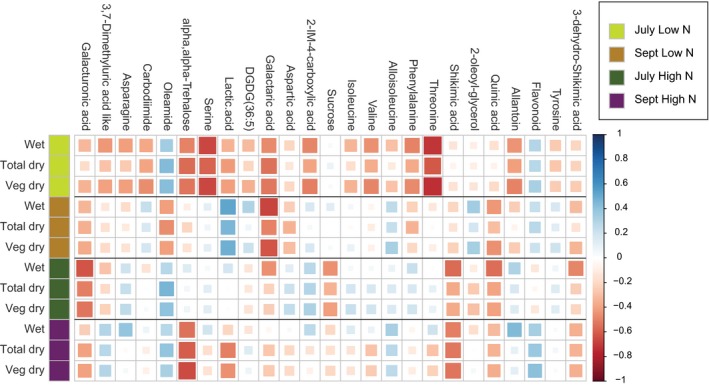
Heatmap showing spearman rank correlations of agronomic traits (rows) and root metabolites (columns). Color scale for correlation value is dark blue: *R* ^2^ = 1; dark red (strong positive association): *R* ^2^ = −1 (strong negative association). Squares are also sized according to *R* ^2^ values with larger squares indicating values close to 1 (blue) or −1 (red). Rows are grouped by collection date (July or September) and treatment (low or full N) with a colored key along the left edge as shown in the legend. Agronomic traits are abbreviated as: wet = total plant (includes stems, leaves, and panicle) fresh weight, total dry = total plant (includes stems, leaves, and panicle) dry matter weight, veg dry = vegetative portion of plant (stems and leaves) dry weight measured in kilograms per hectare

The altered metabolite profile with N stress allows for the discovery of associations with agronomic traits, for example, biomass. Spearman rank correlations (Sheskin, 2003; Weatherburn, 1961) of root metabolites with these traits, arranged by sampling date and N treatment, revealed that metabolite associations with biomass differed with N treatment and also changed over the growing season (Figure 2). Under low N conditions, the July sampling revealed many root metabolites that were correlated with reduced biomass including several proteinogenic amino acids: serine, threonine, asparagine, valine, and phenylalanine. Oleamide, an amide derivative of oleic acid, was associated with higher biomass under full N for both sampling dates and under high N in September. However, Oleamide was negatively correlated with biomass in September under low N conditions. Higher biomass in the low N field was associated with root lactic acid, an end product of anaerobic respiration (Rivoal & Hanson, 1994), but only for the September sampling. Roots with reduced shikimic acid, a precursor in aromatic amino acid biosynthesis, were also associated with higher biomass under full N conditions for both sampling dates, but this correlation with biomass was very weak in the low N treatment. Similarly, reduced quinic acid, another precursor in aromatic amino acid biosynthesis, was associated with higher biomass in roots grown under full N conditions for both sampling dates and low N September sampling, but this association was very weak for the July sampling under low N conditions. When considering differences between July and September sampling dates, it is important to note that sorghum plants were in the vegetative stage in July and were in the reproductive stage in September which likely influenced differences observed between sampling dates. Thus, with the data at hand it is not possible to separate the influence of developmental stage from environmental factors. Galacturonic acid, a component of pectin that makes up plant cell walls, was the only metabolite negatively associated with biomass across all treatment groups and sample dates. No root metabolites were consistently associated with higher biomass across all treatments and time points.

### 3.2 Rhizosphere microbiota significantly vary with N treatment and collection date

**Figure 3:**
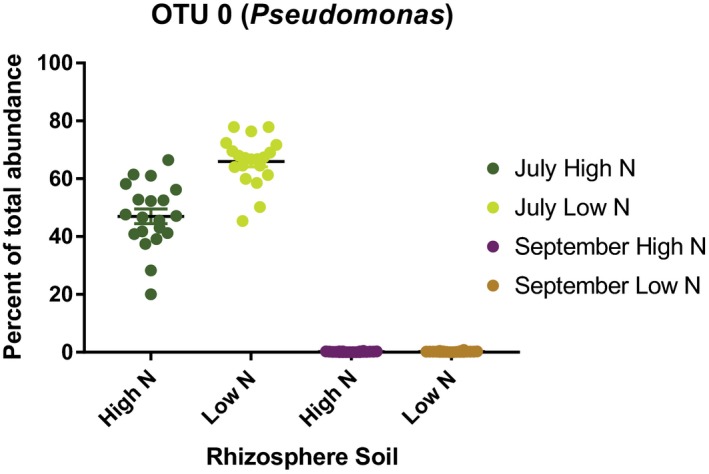
OTU 0 (*Pseudomonas*) dominated the rhizosphere under both high and low N conditions, but was significantly more abundant under low N conditions (*p* < 0.05, ANOVA). Boxplot of OTU 0 (*Pseudomonas*) shown as percent abundance of total normalized reads in rhizosphere soil from the July sampling and demonstrates the dominance of the rhizosphere community by OTU 0

Rhizosphere bacterial community profiles significantly differed according to collection date (PERMANOVA: *R* ^2^ = 0.83, *p* < 0.01) and were clearly separated in a principal coordinate analysis (PCoA; Supporting Information Figure S2). While date accounted for the majority of the variation in rhizosphere bacterial composition, significant variation due to N treatment was also seen along PCo2 explaining 6% of the variation. PERMANOVA analysis revealed that the rhizosphere bacterial community profile was significantly different between low versus full N in both July and September (*p* = 0.001). All of these genera were significantly different by interaction of date and treatment and all except for the Burkholderia genus significantly differed by date (Supporting Information Table S2). The bacterial composition of the rhizosphere was largely dominated by a single operational taxonomic unit (OTU), OTU 0, in July that mapped taxonomically to the *Pseudomonas* genus. In July, this single OTU dominated the rhizosphere, comprising 47% of the bacterial community under full N conditions and significantly more under low N at 66% of the bacterial community (ANOVA, *p* < 0.05; Figure 3).

### 3.3 Metabolic pathway analysis suggests altered flux through shikimate pathway

**Figure 4:**
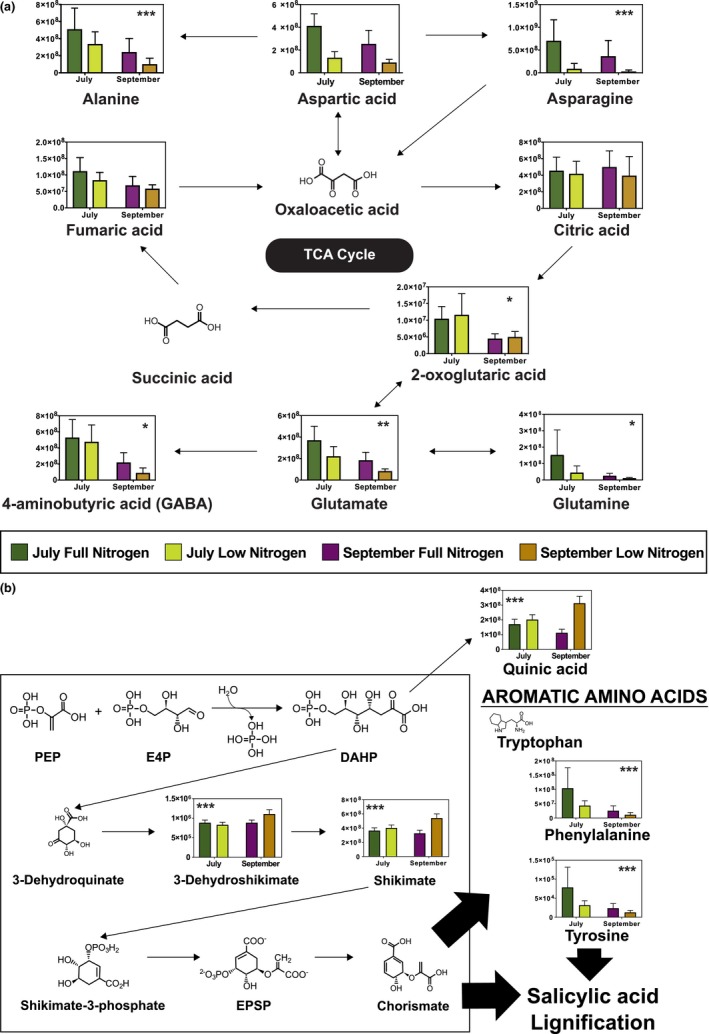
Pathway analysis (a) alanine, aspartate, and glutamate metabolism and (b) phenylalanine, tyrosine, and tryptophan biosynthesis (shikimate pathway). Metabolites detected during metabolomics analysis have peak intensities presented as bar graphs overlaid on the pathway map. Peak intensity reflects the semiquantitative nature of the nontargeted approach used for this global metabolite analysis. Statistical significance when using a nonparametric factorial ANOVA test (Supporting Information Table S2) is denoted as follows: *significant by date, **significant by treatment and date but not the interaction, ***significant by date treatment interaction (*p *<* *0.05). Dark green = July high N; Dark purple = September high N; Light green = July low N; Light purple = September low N

The complete annotated list of metabolites detected in roots from the July sampling was used to generate the metabolic pathways associated with these molecules using the pathway analysis tool in MetaboAnalyst (http://www.metaboanalyst.ca/). The list of pathways matching with the highest number of metabolites in the experimental dataset, without consideration of which metabolites are most relevant to experimental factors, is shown in Supporting Information Table S1 and was used to prioritize KEGG reference pathways of interest. Metabolites that differentiated between low and full N treatments were identified through the PCA biplot analysis (Figure 1) to narrow pathways of interest. Two of these KEGG pathways, “*Alanine, aspartate, and glutamate metabolism*” and “*Phenylalanine, tyrosine, and tryptophan biosynthesis*”, contained metabolites shown to discriminate between low and full N treatment groups and were selected for further analysis. Generally, metabolites in the *“Alanine, aspartate, and glutamate”* pathway were less abundant in roots under N stress, consistent with decreased N availability (Figure 4a, Supporting Information Table S3). Biosynthesis of the aromatic amino acids phenylalanine, tyrosine, and tryptophan occurs via the shikimate pathway. In roots sampled in July, three intermediary metabolites in this pathway, quinic acid, 3‐dehydroshikimic acid, and shikimic acid, were more abundant under N stress, while two end products of this pathway, phenylalanine and tyrosine, were less abundant with N stress (Figure 4b, Supporting Information Table S3).

### 3.4 N stress and the sorghum defense response

**Figure 5:**
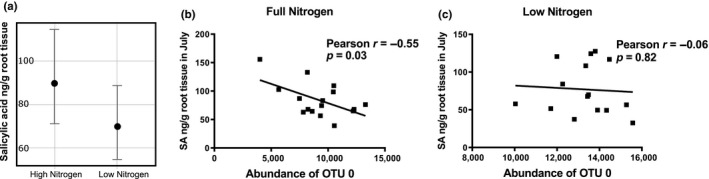
Salicylic acid and OTU 0 abundance. The main effect of nitrogen treatment showed significantly reduced root salicylic acid content under low N compared to high N when averaged over the two sampling dates and treating genotype as a random effect. Panel (a) shows the effects plot with 95% confidence interval for the linear mixed model, (b) OTU 0 is negatively correlated with salicylic acid with full N and (c) not with low N

Analysis of phytohormones in root tissue was performed and included quantitative measurement of 12‐oxo‐phytodienoic acid, trans‐zeatin riboside, jasmonic acid, salicylic acid, abscisic acid, phaseic acid, indole‐3‐carboxylic acid, and dihydrophaseic acid. When treating genotype as a random factor, 12‐oxo‐phytodienoic acid, jasmonic acid, and trans‐zeatin riboside significantly varied in the September root sampling as compared to roots sampled in July, but did not vary significantly by treatment or by interaction of date and treatment (Supporting Information Table S4 and Data S1). Salicylic acid was significantly reduced in low N conditions (Figure 5a), but did not vary significantly by date or by date x treatment interaction (Supporting Information Table S4). No other root phytohormones that were analyzed were significantly altered by N treatment. Furthermore, for roots sampled in July, less SA content was correlated with greater abundance of the rhizosphere‐dominating OTU 0 (*Pseudomonas)* under full N (Figure 5b) but not under low N conditions (Figure 5c). To further investigate root defense response, orthologs of genes previously described as having altered expression during pathogenesis (van Loon, Rep, & Pieterse, 2006) were investigated using RNA‐seq data generated from roots sampled in parallel from the same field and dates as the root metabolite collections (Supporting Information Figure S4). The RNA‐seq data were generated from only a subset of genotypes (PI 297130, PI 655972, CO53, CO56, and C225) with little replication (two biological replicates), which limited the statistical power. However, three of the 12 pathogenesis‐related (PR) genes (PR1b, OsPR8, and OsPR10b) listed were downregulated in roots collected in July under low N versus full N conditions in multiple genotypes (Students *t* test, *p* < 0.01; Supporting Information Figure S4). Expression of PR1b was reduced by 90% (on average) and was downregulated in PI 655972, PI 297130, CO56, and C225. Expression of OsPR8 was reduced by 60% (on average) and was downregulated in PI 297130, CO56, and C225. Expression of OsPR10b was also reduced by 60% (on average) and was downregulated in all included genotypes. No significant difference in low N versus full N gene expression for PR1b, OsPR8, or OsPR10b was observed, however, the limited number of replicates severely restricted statistical power.

## 4 DISCUSSION

Root and rhizosphere soil samples from N stressed sorghum revealed differences in root metabolite profiles, rhizosphere microbial community composition, and SA production. Phytohormones and bacterial community composition also varied over the growing season. Metabolites that accumulated in N stressed roots are consistent with previous studies on the effects of nutrient deprivation on plant metabolism and go beyond what is currently known. In the July sampling, for example, we observed increased trehalose and sucrose content in roots with low N relative to full N. Similarly, an increased emphasis on carbohydrate storage in roots of soybean plants has been previously associated with a limited ability to synthesize sucrose in leaves with N stress (Rufty, Huber, & Volk, 1988). Enhanced storage of carbohydrates in roots versus leaves could explain the observed increase in trehalose and sucrose in roots with low N relative to full N. However, trehalose accumulation has been associated with both biotic and abiotic plant stress response (Fernandez, Béthencourt, Quero, Sangwan, & Clément, 2010). Accumulation of trehalose in roots was observed in *Arabidopsis thaliana* infected with *Plasmodiophora brassicae* (Brodmann et al., 2002) and in *Pinus sylvestris* (pine) infected with *Armillaria ostoyae* (Isidorov, Lech, Żółciak, Rusak, & Szczepaniak, 2008). Therefore, it is possible that trehalose accumulation in roots of N stressed sorghum resulted from the combined abiotic and biotic stresses of low N and the proliferation of *Pseudomonas* in the rhizosphere in July.

In addition, metabolites identified in previous research as important to N cycling and mobility in plants were more abundant in roots grown with full N relative to low N. For example, both aspartate and asparagine function were reduced with low N and act as carriers when mobilizing N to sink tissues with asparagine being particularly important as it is efficiently transported (Cañas, Quilleré, Lea, & Hirel, 2010; Gaufichon, Rothstein, & Suzuki, 2015; Masclaux‐Daubresse et al., 2010). In other studies looking at N stress, reduced amino acids were observed in both root exudates (Carvalhais et al., 2011) and tomato root tissue (Sung et al., 2015). Thus, increased mobilization of N can reflect higher availability of soil N. However, it is also possible that this increased mobilization of N is related to competition with soil microbes because access to N is important not only to plants, but also to pathogenic and beneficial microorganisms living in association with plants. While N application to crops likely benefits plant defense, it may also allow rhizosphere microorganisms to gain access to N coming from root cellular pools (Hoffland, Jeger, & van Beusichem, 2000; Jensen & Munk, 1997). Gene expression patterns consistent with remobilization of N as a strategy to sequester N stores away from bacteria have been reported previously in *Phaseolus vulgaris* (common bean) (Tavernier et al., 2007), tomato (Olea et al., 2004), *Nicotiana tabacum* L. (tobacco) (Pageau, Reisdorf‐Cren, Morot‐Gaudry, & Masclaux‐Daubresse, 2005), and *Arabidopsis thaliana* (AbuQamar et al., 2006). Glutamine is typically the preferred carrier during N mobilization, but asparagine, aspartate, or alanine may be utilized when glutamine is limited (Pellier, Laugé, Veneault‐Fourrey, & Langin, 2003). A decrease in N‐carrying amino acids in response to N stress likely reflects lower N stores already present in plant cells. Pathogens, as well as other soil microbes, may have experienced reduced opportunity for N exploitation in roots of N stressed sorghum.

Nitrogen stressed sorghum showed unique metabolic associations with agronomic traits that changed over the growing season. Previous research with non‐stressed, greenhouse‐grown sorghum revealed that higher levels of intermediaries of the shikimate pathway, quinic, and shikimic acids, in 4‐week‐old leaves was associated with higher biomass (Turner et al., 2016). However, in the current work, lower content of quinic and shikimic acids was associated with higher biomass in roots under full N conditions. Shikimic acid was not strongly correlated with biomass under N stress but reduced 3‐dehydroshikimic acid, a related intermediary in the shikimate pathway, was negatively correlated with higher biomass. Lower quinic acid content was also correlated with higher biomass under low N conditions, but only in roots from the September sampling. Whether this discrepancy is due to greenhouse versus field conditions or reflects tissue‐specific effects is not clear (Turner et al., 2016). Therefore, the role of shikimate pathway metabolites and biomass accumulation, particularly in N stress environments warrants further investigation.

The metabolic pathway analysis suggested altered flux through the shikimate pathway that may have impaired production of the plant defense hormone SA. An increase in intermediary metabolites of this pathway combined with a decrease in end products with N stress suggests that flux through the shikimate pathway may have been compromised, or compounds may have been diverted to other biosynthetic pathways, resulting in lower concentrations of end products (Figure 4b, Supporting Information Table S3). The end products of the shikimate pathway are aromatic amino acids, including phenylalanine, which is used in the biosynthesis of plant defense hormone SA (Chen, Zheng, Huang, Lai, & Fan, 2009; Maeda, Yoo, & Dudareva, 2011). Our results show that SA concentration was significantly lower in roots experiencing N stress and this effect was not influenced by collection date when treating genotype as a random effect in a mixed linear model analysis (Figure 5a, Supporting Information Table S4). Since SA plays an important role in plant immunity and defense, reduced metabolic capacity to produce SA may alter the overall plant defense response.

RNA‐seq analysis of a subset of root samples for sorghum genes involved in the pathogenesis response provided some evidence of altered expression with three of the 12 genes being downregulated (*t* test, *p* = 0.01) in samples from multiple genotypes (Supporting Information Figure S2). Homologs in *Arabidopsis thaliana* for two of these genes, PR1b, were previously found to be induced in response to SA (Thomma et al., 1998). In sorghum, PR1b and other PR genes were also induced when SA was added to growth solution for hydroponically grown plants (Salzman et al., 2005). Furthermore, sorghum grown under full N conditions was able to accumulate more root SA and also had lower abundance of the rhizosphere‐dominating OTU 0 (*Pseudomonas)* (Figure 5b) consistent with successful plant defense of the rhizosphere. However, sorghum experiencing N stress had less SA accumulation in root tissue and did not show any reduction in rhizosphere abundance of OTU 0 (*Pseudomonas)* (Figure 5c). These results suggest that the reduced abundance of SA in roots experiencing N stress is insufficient to induce some important defense genes, which is also supported by the RNA‐seq analysis (Supporting Information Figure S4). However, since bacterial composition in the rhizosphere also varied significantly under low N conditions (Supporting Information Table S2), we cannot determine conclusively if the reduced abundance of SA was due to metabolic effects of N stress or soil bacterial interactions. When interpreting both metabolic and microbial differences due to treatment effect, it is important to note other differences between fields. The low N field utilized in this study, which had not had N applied for more than 20 years, also had other differences relative to the full N field including crop rotation, soil composition and likely others. However, 78.8% of variation in the rhizosphere bacterial composition was explained by sampling date and only 5.7% variation was due to different treatments/fields (Supporting Information Figure S3). Similarly, when metabolite data were plotted together in PCA analysis, separation by date along PC1 explained 49.3% of variation and PC2 explained only 13.4%. These results suggest that the influence of non‐field specific factors such as plant growth and developmental stage on the soil rhizosphere community composition are exerting a stronger effect on sample variation than any potential effects due to bulk soil composition, which has also been demonstrated in other research (Shi et al., 2015).

Identifying production strategies that incorporate marginal soils with reduced inputs is a high priority for biofuels research and our findings revealed important implications for improving biomass yield under N stress and in the context of microbial interactions. As breeding programs are established for biofuels production on marginal lands, priorities should be established for both abiotic and biotic stress tolerance since plant response to N deficiency is likely to affect critical metabolic pathways for plant defense.

## 5 ACCESSION NUMBERS

Sequence data from this article can be found in the NCBI SRA submission library under the following accession numbers:

1. 16S amplicons: Sequencing project IDs #1095844, #1095845, #1095846; SRA Identifier #SRP165130.
2. RNA‐seq sequences: Submission #SUB4139833, SRA Identifier #SRP151132.

## CONFLICT OF INTEREST

The authors have no conflict of interest to declare for this research.

## AUTHOR CONTRIBUTIONS

DPS, TB, ALE, JEP, ST, SK, and PL contributed to the research design. SK provided sorghum seed and DPS and ELM performed field experiments. ELM, IK, DC, MB, and AMS performed various laboratory experiments. CY, EG, DC, IK, and PL analyzed the results with AMS. AMS interpreted the data and wrote the paper with contributions from DC, IK, ST, ALE, ELM, DPS, SK, CY, EG, PL, and JEP.
