# Supplementary material for: Metabolomics of sorghum roots during nitrogen stress reveals compromised metabolic capacity for salicylic acid biosynthesis
Source: Plant Direct. 2019 Mar 14;3(3):e00122. doi: 10.1002/pld3.122 (PMC6508800; doi:10.1002/pld3.122)
Supplement: Supplementary file 1 [file PLD3-3-e00122-s001.docx]

**Figure S1.** Scatter plot shows fold change in total above ground dry biomass originally measured in kilograms per hectare (kg/ha). The mean is represented as a bar and whiskers represent the standard error of the mean. No significant difference in biomass reduction under low N (low N kg/ha : full N kg/ha) was identified via the student’s t-test comparison of hybrids and inbreds.

**
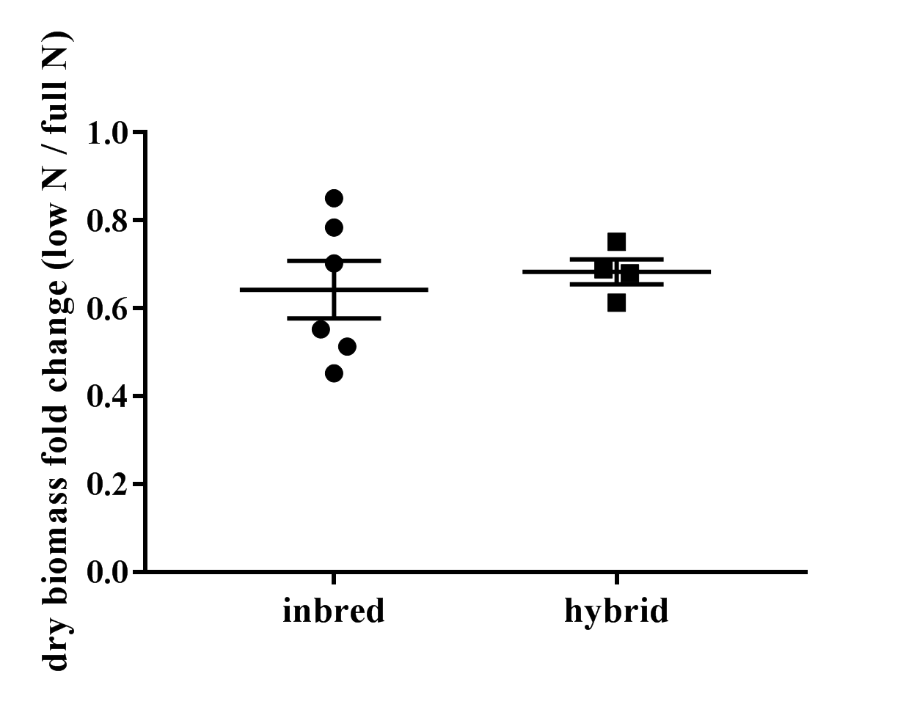
**

| **Genotype** | **Breed** | **Dry above ground biomass fold change (low N / full N)** |
| --- | --- | --- |
| C225 | hybrid | 0.75 |
| C053 | hybrid | 0.61 |
| C056 | hybrid | 0.69 |
| C126 | hybrid | 0.68 |
| PI 297130 | inbred | 0.70 |
| PI 505735 | inbred | 0.51 |
| PI 506030 | inbred | 0.78 |
| PI 510757 | inbred | 0.85 |
| PI 642998 | inbred | 0.55 |
| PI 655972 | inbred | 0.45 |
