# Supplementary material for: Metabolomics of sorghum roots during nitrogen stress reveals compromised metabolic capacity for salicylic acid biosynthesis
Source: Plant Direct. 2019 Mar 14;3(3):e00122. doi: 10.1002/pld3.122 (PMC6508800; doi:10.1002/pld3.122)
Supplement: Supplementary file 2 [file PLD3-3-e00122-s002.docx]

**Figure S2.** Bar plot shows the relative abundance of the 30 most abundant genera (Table S2) in the sorghum rhizosphere. Bars are organized by sampling date, which explains 78.8% of bacterial rhizosphere composition (Figure S3). Whitespace represents the percent of sequences that were not classified to one of the 30 most abundant genera. * = significant by date and date x treatment interaction, ** = significant by treatment and date interaction only.

| 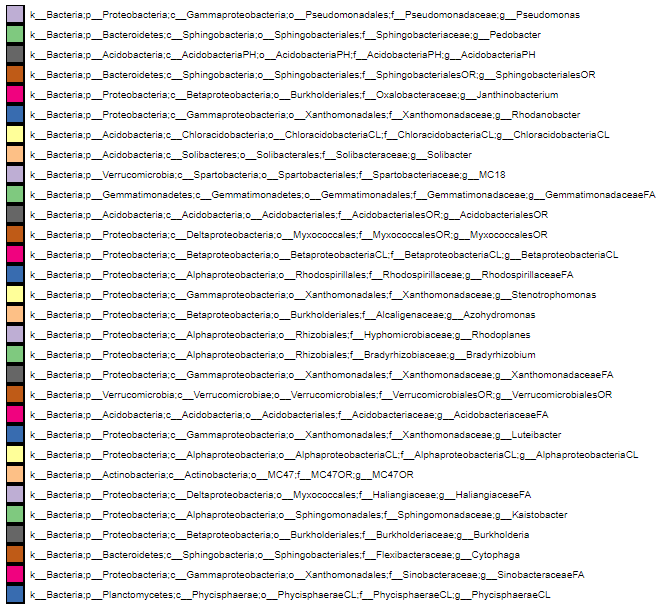 | Pseudomonas*  Pedobacter*  AcidobacteriaPH*  SphingobacterialesOR*  Janthinobacterium*  Rhodanobacter*  ChloracidobacteriaCL*  Solibacter*  MC18*  GemmatimonadaceaeFA*  AcidobacterialesOR*  MyxococcalesOR*  BetaproteobacteriaCL*  RhodospirillaceaeFA*  Stenotrophomonas*  Azohydromonas*  Rhodoplanes*  Bradyrhizobium*  XanthomonadaceaeFA*  VerrucomicrobialesOR*  AcidobacteriaceaeFA*  Luteibacter*  AlphaproteobacteriaCL*  MC47OR*  HaliangiaceaeFA*  Kaistobacter*  Burkholderia**  Cytophaga*  SinobacteraceaeFA*  PhycisphaeraeCL* | 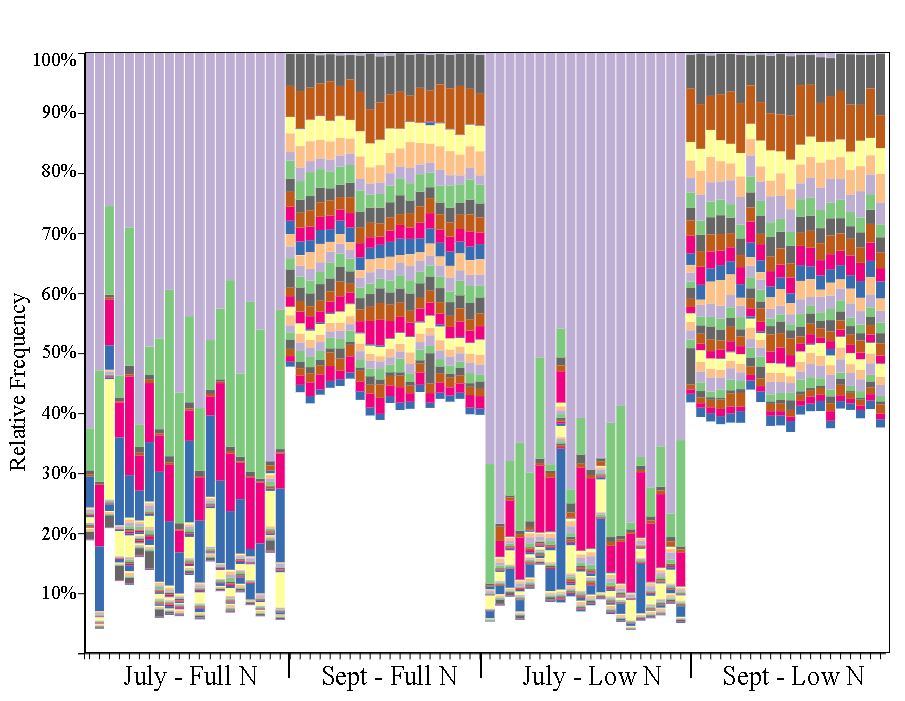 |
| --- | --- | --- |
