# Supplementary material for: Metabolomics of sorghum roots during nitrogen stress reveals compromised metabolic capacity for salicylic acid biosynthesis
Source: Plant Direct. 2019 Mar 14;3(3):e00122. doi: 10.1002/pld3.122 (PMC6508800; doi:10.1002/pld3.122)
Supplement: Supplementary file 3 [file PLD3-3-e00122-s003.docx]

**Figure S3.**  Scores plot from PCoA of the 16S V4 amplicon data from rhizosphere soil at the OTU level using the Bray-Curtis Index as a distance method (PERMANOVA R^2^=0.83, p < 0.01).

**
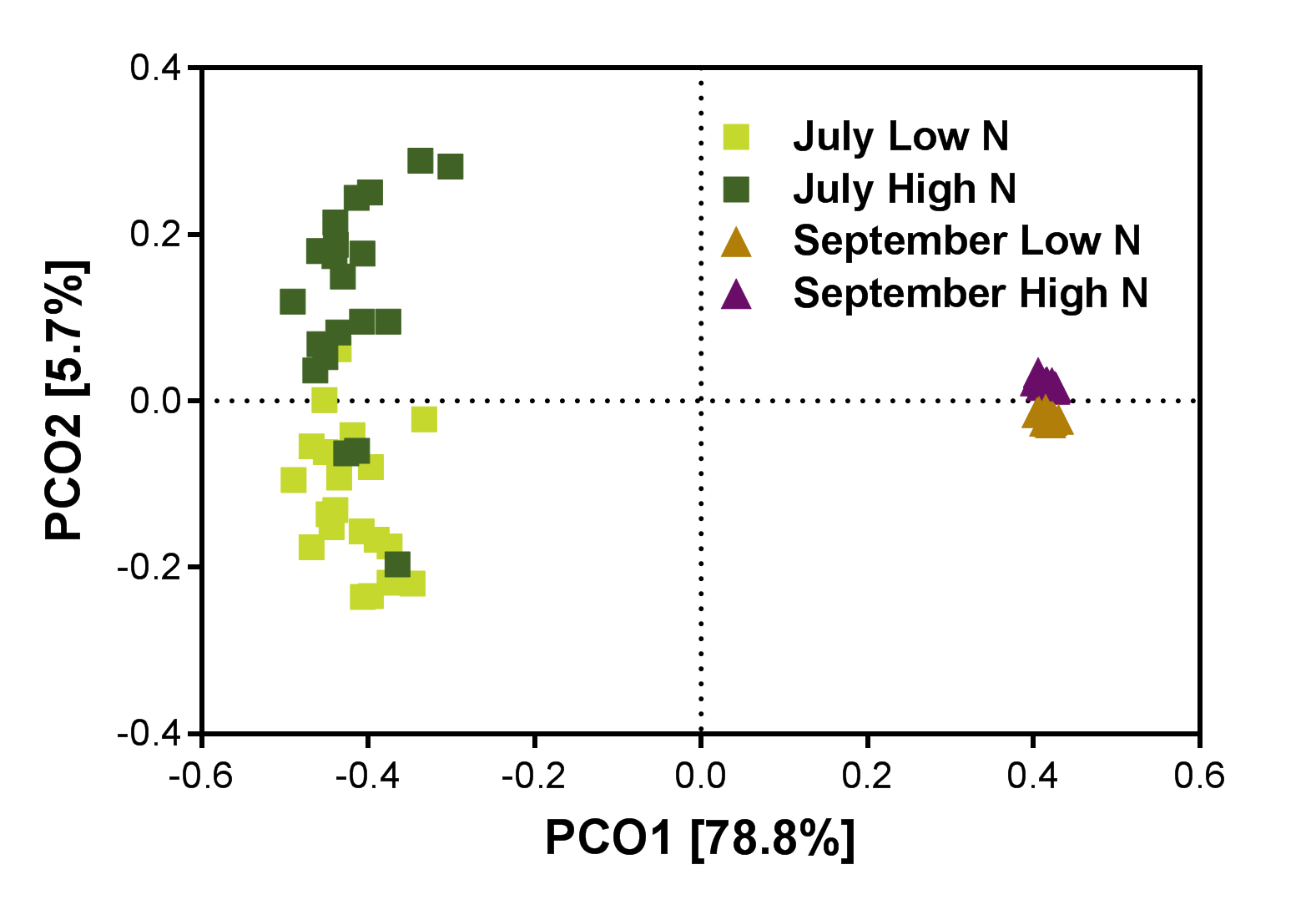
**
