# Supplementary material for: Metabolomics of sorghum roots during nitrogen stress reveals compromised metabolic capacity for salicylic acid biosynthesis
Source: Plant Direct. 2019 Mar 14;3(3):e00122. doi: 10.1002/pld3.122 (PMC6508800; doi:10.1002/pld3.122)
Supplement: Supplementary file 4 [file PLD3-3-e00122-s004.docx]

**Figure S4 –** Heatmap shows z-scores of RNA-seq expression data for putative Sorghum orthologs of twelve previously described pathogenesis-related genes. Samples are displayed as columns (N = 14) and genes as rows and both are clustered according to similar expression patterns. Blue/red indicates higher/lower gene expression relative to the average expression across the sample set. Sample names indicate date (Jul = July; Sep = September), treatment (Lo = low N; Hi = full N), and genotype (N – 1: PI_297130, N – 2: PI_655972, N – 3: CO53, N – 4: CO56, N – 5: C225). Gene abbreviations, top BlastP hit, and probable function are included in the table below the figure. Genes with statistically reduced expression in low N versus full N in July using a student’s t-test are indicated with an asterisk (*).

z-score


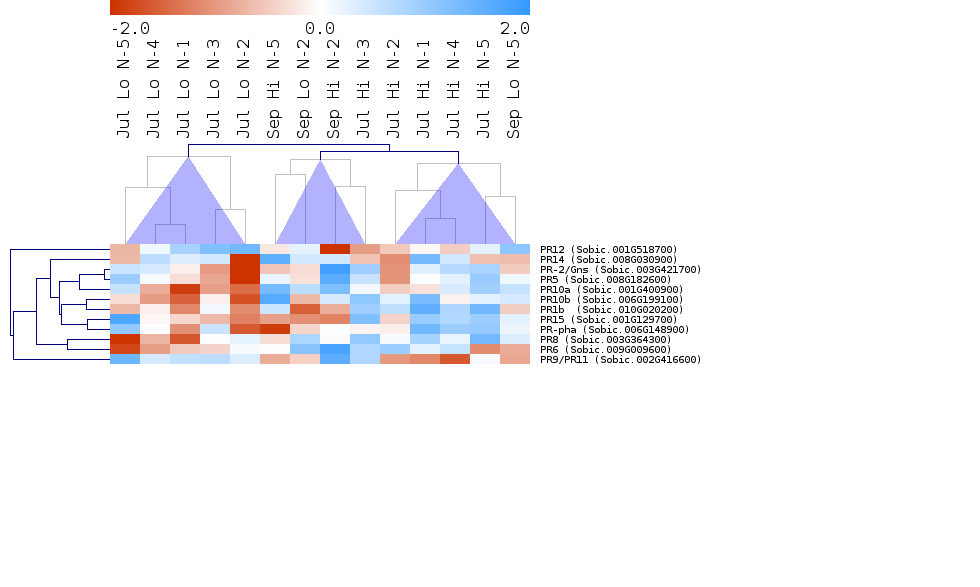


| **Gene** | **Top BlastP Hit** | **Probable function** |
| --- | --- | --- |
| PR1b* | Sobic.010G020200 | defense-related protein containing SCP domain |
| PR-2/Gns | Sobic.003G421700 | Glucan endo-1,3-beta-D-glucosidase |
| OsPR5 | Sobic.008G182600 | thaumitin-like protein |
| OsPR6 | Sobic.009G009600 | cystatin, protein-inhibitor |
| OsPR8* | Sobic.003G364300 | chitinase |
| OsPR9 | Sobic.002G416600 | peroxidase |
| OsPR10a | Sobic.001G400900 | RNAse |
| OsPR10b* | Sobic.006G199100 | (S)-norcoclaurine synthase |
| OsPR12 | Sobic.001G518700 | Gamma-thionin family |
| OsPR14 | Sobic.008G030900 | Lipid transfer protein |
| OsPR15 | Sobic.001G129700 | Uncharacterized, contains cupin domain |
| PR-pha | Sobic.006G148900 | Phenylalanine ammonia lyase |
