# Supplementary material for: Metabolomics of sorghum roots during nitrogen stress reveals compromised metabolic capacity for salicylic acid biosynthesis
Source: Plant Direct. 2019 Mar 14;3(3):e00122. doi: 10.1002/pld3.122 (PMC6508800; doi:10.1002/pld3.122)

**Figure S5.** A schematic presentation of the root tissue extraction technique for metabolomics analysis.


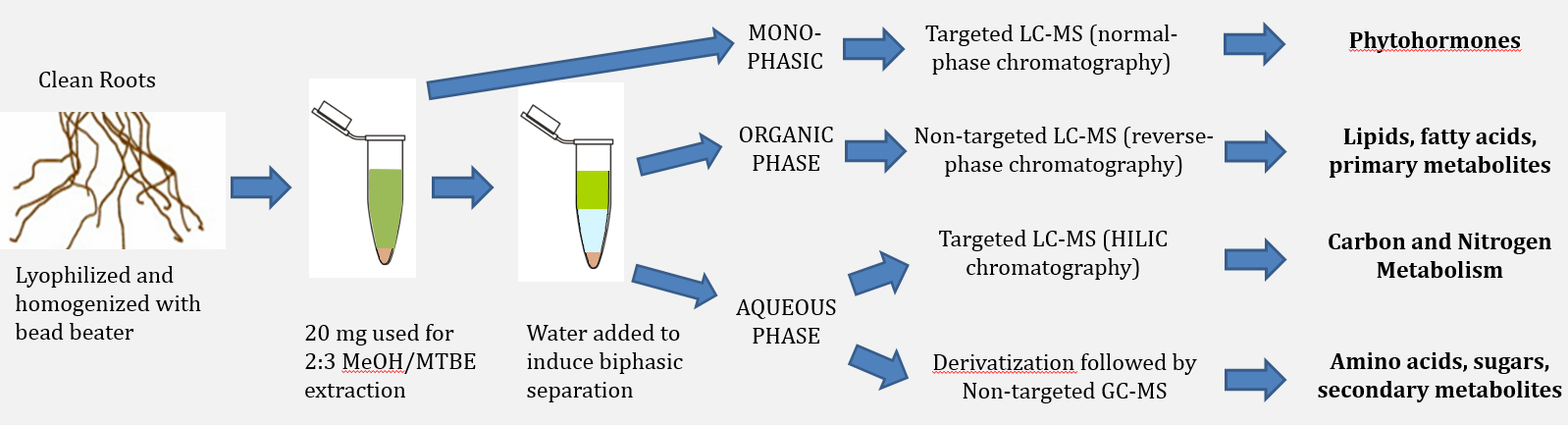

Supplement: Supplementary file 5 [file PLD3-3-e00122-s005.docx]
