# Supplementary material for: Metabolomics of sorghum roots during nitrogen stress reveals compromised metabolic capacity for salicylic acid biosynthesis
Source: Plant Direct. 2019 Mar 14;3(3):e00122. doi: 10.1002/pld3.122 (PMC6508800; doi:10.1002/pld3.122)
Supplement: Supplementary file 6 [file PLD3-3-e00122-s006.docx]

**Table S1.** Pathway analysis of root metabolites using MetaboAnalyst's Pathway Analysis (n = 34). Pathway name = KEGG metabolic pathway, ‘Match Status’ reflects how many metabolites were present in the dataset over total number of metabolites in the pathway, p-value = using Fisher’s exact test, FDR corrects the p-value for false discovery rate using Benjamini Hochberg, ‘Impact’ sums the importance of each matching metabolite node (node centrality measures normalized to total importance of the pathway; maximum = 1) as a cumulative percentage. Pathways with significant p-values (p < 0.05) were significantly impacted by nitrogen stress based upon pathway topology analysis. The pathway analysis in Metaboanalyst has previously been described in full detail (Xia *et al.*, 2015).

| **Pathway Name** | **Match Status** | **p-value** | **FDR** | **Impact** |
| --- | --- | --- | --- | --- |
| Aminoacyl-tRNA biosynthesis | 17/67 | 4.06E-06 | 3.37E-04 | 0.09302 |
| Alanine, aspartate and glutamate metabolism | 8/21 | 8.31E-05 | 0.003449 | 0.84932 |
| Cyanoamino acid metabolism | 4/11 | 0.00726 | 0.20086 | 1 |
| Galactose metabolism | 6/26 | 0.011788 | 0.2446 | 0.1146 |
| Ascorbate and aldarate metabolism | 4/14 | 0.018389 | 0.26985 | 0 |
| Arginine and proline metabolism | 7/37 | 0.019821 | 0.26985 | 0.27451 |
| Phenylalanine, tyrosine and tryptophan biosynthesis | 5/22 | 0.022758 | 0.26985 | 0.1794 |
| Nitrogen (N) metabolism | 4/16 | 0.029669 | 0.30781 | 0 |
