# Supplementary material for: Metabolomics of sorghum roots during nitrogen stress reveals compromised metabolic capacity for salicylic acid biosynthesis
Source: Plant Direct. 2019 Mar 14;3(3):e00122. doi: 10.1002/pld3.122 (PMC6508800; doi:10.1002/pld3.122)
Supplement: Supplementary file 7 [file PLD3-3-e00122-s007.docx]

**Table S2.** Microbial composition as relative abundance of the top 30 most abundant Genera in the rhizosphere (means and results of ANCOM test in QIIME2 by trait) (n=20). Statistical significance when using the ANCOM test is denoted as follows: * = significant by date and date x treatment interaction, ** = significant by treatment and date interaction only.
