# Supplementary material for: Metabolomics of sorghum roots during nitrogen stress reveals compromised metabolic capacity for salicylic acid biosynthesis
Source: Plant Direct. 2019 Mar 14;3(3):e00122. doi: 10.1002/pld3.122 (PMC6508800; doi:10.1002/pld3.122)
Supplement: Supplementary file 8 [file PLD3-3-e00122-s008.docx]

**Table S3.** Effects of N treatment and harvest date on root metabolite content as determined by linear mixed model analysis.

| **Factor** | **Nitrogen (N)** | | **Date** | | **Date * N** | |
| --- | --- | --- | --- | --- | --- | --- |
| **Metabolite** | *F* | *P* | *F* | *P* | *F* | *P* |
| 2-Imidazolidone-4-carboxylic acid | 68.49 | 0.017* | 21.68 | 0* | 1.131 | 0.2924 |
| 2-oxo-glutaric acid | 1.065 | 0.4188 | 54.02 | 0* | 54.51 | 0.7754 |
| 3-dehydro-shikimic acid | 10.55 | 0.0932 | 1.709 | 0.1967 | 14.11 | 0.0004* |
| 3,7-Dimethyl uric acid (similar to) | 90.4 | 0.0143* | 27.18 | 0* | 54.53 | 0.0138* |
| 4-aminobutyric acid | 3.727 | 0.2047 | 82.01 | 0* | 0.8128 | 0.3712 |
| Alanine | 15.31 | 0.0683 | 42.76 | 0* | 0.6457 | 0.4252 |
| Allantoin | 29.75 | 0.0326* | 28.31 | 0* | 13.49 | 0.0005* |
| Alpha-alpha-trehalose | 4.714 | 0.1735 | 4.355 | 0.0417* | 7.838 | 0.0071* |
| Asparagine | 47.43 | 0.0255* | 22.25 | 0* | 13.45 | 0.0006* |
| Aspartic acid | 88.63 | 0.0128* | 24.09 | 0* | 1.85 | 0.1795 |
| Carbodiimide | 40.28 | 0.0295* | 90.73 | 0* | 3.435 | 0.0693 |
| Citric acid | 0.908 | 0.4428 | 0.07983 | 0.7786 | 0.5202 | 0.4738 |
| DGDG(36:5) | 48.65 | 0.0249* | 0.2058 | 0.6519 | 6.532 | 0.0135* |
| Fumaric acid | 2.463 | 0.268 | 3.194 | 0.0795 | 0.006961 | 0.9338 |
| Galactaric acid | 0.3215 | 0.6285 | 5.108 | 0.0279 | 6.584 | 0.0131 |
| Galacturonic acid | 0.0059 | 0.9389 | 13.66 | 0.0005* | 5.954 | 0.0179 |
| Glutamine | 12.81 | 0.0737 | 41.9 | 0* | 14.02 | 0.0004* |
| Glutamate | 20.33 | 0.0484* | 78.02 | 0* | 1.781 | 0.1875 |
| Lactic acid | 6.448 | 0.1348 | 21.4 | 0* | 9.431 | 0.0033* |
| Flavonoid | 44.77 | 0.0269* | 28.06 | 0* | 21.47 | 0* |
| 2-oleoyl-glycerol | 17.25 | 0.0617 | 37.25 | 0* | 4.28 | 0.0434* |
| Alloisoleucine | 29.42 | 0* | 90.6 | 0* | 0.7005 | 0.4062 |
| Isoleucine | 33.77 | 0* | 111.7 | 0* | 0.0258 | 0.873 |
| Oleamide | 0.0767 | 0.8083 | 5.877 | 0.0187* | 54.21 | 0.0143* |
| Phenylalanine | 19.1 | 0.0566 | 41.47 | 0* | 9.848 | 0.0027* |
| Quinic acid | 15.82 | 0.0002* | 0.3636 | 0.549 | 12.03 | 0.001* |
| Serine | 27.01 | 0.039* | 104.6 | 0* | 0.0026 | 0.9595 |
| Shikimic acid | 15.03 | 0.0003* | 0.0067 | 0.935 | 9.797 | 0.0028* |
| Sucrose | 3.591 | 0.0632 | 21.29 | 0* | 4.295 | 0.0429* |
| Threonine | 35.13 | 0.0329* | 84.08 | 0* | 0.3879 | 0.536 |
| Tyrosine | 13.49 | 0.0679 | 59.1 | 0* | 12.24 | 0.0009* |
| Valine | 33.16 | 0.032* | 109.4 | 0* | 0.1356 | 0.7141 |

Results of statistical modeling of non-transformed root metabolite content (relative peak area) as a function of N treatment, harvest date and the interaction between treatment and date. Treatment and date are treated as fixed effects and genotype and biological replicates as random effects. Significance was determined using the F statistic (*F*) and * denotes a significant pvalue (*P <* 0.05), ^a^ = parametric tests (linear mixed model [40, 41]), ^b^ = non-parametric tests (factorial ANOVA [42]).
