# Supplementary material for: Metabolomics of sorghum roots during nitrogen stress reveals compromised metabolic capacity for salicylic acid biosynthesis
Source: Plant Direct. 2019 Mar 14;3(3):e00122. doi: 10.1002/pld3.122 (PMC6508800; doi:10.1002/pld3.122)
Supplement: Supplementary file 9 [file PLD3-3-e00122-s009.docx]

**Table S4.** Effects of N treatment and harvest date on root phytohormone content as determined by linear mixed model analysis.

|  | **OPDA^a^** | | **tZ-r^a^** | | **JA^a^** | | **ABA^b^** | | **PA^b^** | | **IAA^b^** | | **SA^a^** | | **ICA^b^** | | |
| --- | --- | --- | --- | --- | --- | --- | --- | --- | --- | --- | --- | --- | --- | --- | --- | --- | --- |
| Factor | *F* | *P* | *F* | *P* | *F* | *P* | *F* | *P* | *F* | *P* | *F* | *P* | *F* | *P* | *F* | *P* |  |
| Nitrogen (N) | 0.10 | 0.78 | 1.59 | 0.34 | 1.04 | 0.31 | 15.59 | 0.07 | 1.47 | 0.36 | 1.78 | 0.34 | 5.60 | 0.02 | 10.21 | 0.09 |  |
| Date | 25.03 | 0.00* | 43.97 | 0.00* | 45.70 | 0.00* | 46.39 | 0.00* | 52.23 | 0.00* | 52.63 | 0.11 | 0.08 | 0.78 | 2.24 | 0.14 |  |
| Date * N | 3.20 | 0.08 | 1.06 | 0.31 | 1.12 | 0.30 | 52.53 | 0.00* | 52.2 | 0.32 | 52.69 | 0.55 | 2.10 | 0.15 | 0.11 | 0.74 |  |

Results of statistical modeling of non-transformed root phytohormone content (ng · g^-1^ root tissue) as a function of N treatment, harvest date and the interaction between treatment and date. Treatment and date are treated as fixed effects and genotype and biological replicates as random effects. Significance was determined using the F statistic (*F*) and * denotes a significant pvalue (*P <* 0.05), ^a^ = parametric tests (linear mixed model [40, 41]), ^b^ = non-parametric tests (factorial ANOVA [42]).

12-oxo-phytodienoic acid = OPDA, tZ-r = trans-zeatin riboside, JA = Jasmonic Acid, ABA = Abscisic acid, PA = Phaseic acid, IAA = Indole-3-carboxylic acid, SA = Salicylic acid, ICA = Indole-3-carboxylic acid, DPA = Dihydrophaseic acid
