# Supplementary material for: Metabolomics of sorghum roots during nitrogen stress reveals compromised metabolic capacity for salicylic acid biosynthesis
Source: Plant Direct. 2019 Mar 14;3(3):e00122. doi: 10.1002/pld3.122 (PMC6508800; doi:10.1002/pld3.122)
Supplement: Supplementary file 10 [file PLD3-3-e00122-s010.docx]

**Table S5.** SRM Transitions used to identify phytohormones with UPLC-MS-TQS analysis.

| Ion Name | Precursor m/z | Product m/z | Precursor Charge | Collision Energy | Retention Time |
| --- | --- | --- | --- | --- | --- |
| trans-zeatin riboside | 352 | 220 | +1 | 18 | 4.62 |
| trans-zeatin riboside | 352 | 136 | +1 | 18 | 4.62 |
| trans-zeatin | 220.2 | 136.1 | +1 | 32 | 3.91 |
| trans-zeatin | 220.2 | 119.1 | +1 | 32 | 3.91 |
| trans-zeatin | 221.2 | 137.1 | +1 | 32 | 3.91 |
| SA-d4 | 141.1 | 97 | -1 | 30 | 6.78 |
| SA-d4 | 141.1 | 69 | -1 | 30 | 6.78 |
| SA | 137.1 | 93 | -1 | 30 | 6.78 |
| SA | 137.1 | 65 | -1 | 30 | 6.78 |
| PA-d3 | 282.2 | 171.1 | -1 | 16 | 6.57 |
| PA-d3 | 282.2 | 142.1 | -1 | 16 | 6.57 |
| PA | 279.2 | 205.2 | -1 | 10 | 6.57 |
| PA | 279.2 | 139.1 | -1 | 10 | 6.57 |
| OPDA | 291.1 | 247 | -1 | 20 | 13.07 |
| OPDA | 291.1 | 165 | -1 | 20 | 13.07 |
| meSA | 153.1 | 121.1 | +1 | 16 | 9.96 |
| meJA | 225.2 | 151.1 | +1 | 12 | 11.36 |
| meJA | 225.2 | 133.1 | +1 | 12 | 11.36 |
| JA-d5 | 216.2 | 153.1 | +1 | 12 | 8.77 |
| JA-d5 | 216.2 | 135.1 | +1 | 12 | 8.77 |
| JA | 211.2 | 151.1 | +1 | 12 | 8.77 |
| JA | 211.2 | 133.1 | +1 | 12 | 8.77 |
| ICA | 162.1 | 144.1 | +1 | 10 | 6.37 |
| ICA | 162.1 | 118.1 | +1 | 10 | 6.37 |
| IBA | 204.1 | 186.1 | +1 | 22 | 8.77 |
| IBA | 204.1 | 130.1 | +1 | 22 | 8.77 |
| IAnitrile | 157 | 130 | +1 | 19 | 8.36 |
| IAnitrile | 157 | 117 | +1 | 19 | 8.36 |
| IAcrA | 188.1 | 170.1 | +1 | 22 | 7.67 |
| IAcrA | 188.1 | 115.1 | +1 | 22 | 7.67 |
| IAamide | 175 | 130 | +1 | 28 | 6.94 |
| IAamide | 175 | 103 | +1 | 28 | 6.94 |
| IA-alanine | 247 | 130 | +1 | 20 | 6.5 |
| IA-alanine | 247 | 90 | +1 | 20 | 6.5 |
| IAA-d5 | 181.1 | 134.1 | +1 | 30 | 7.07 |
