# Supplementary material for: Metabolomics of sorghum roots during nitrogen stress reveals compromised metabolic capacity for salicylic acid biosynthesis
Source: Plant Direct. 2019 Mar 14;3(3):e00122. doi: 10.1002/pld3.122 (PMC6508800; doi:10.1002/pld3.122)
Supplement: Supplementary file 12 [file PLD3-3-e00122-s012.pdf]

Response to Editor Comments

1. The clean copy contains an introductory paragraph at the beginning of the Results section that is not present in the marked up copy of the manuscript. The authors should be sure that the correct "clean" files have been submitted for publication.

    The introductory paragraph at the beginning of the results section was present but somehow the section heading "Low N impacts on plant biomass accumulation" was missing in the clean version. This has been corrected.

2. Specific comments:
Line 186: To what file does Supplemental Data 2 refer? Only one Supplemental Data file could be identified.

The supplemental data was originally separated into multiple files but ultimately combined into a single file with multiple tabs for submission. The "supplemental data 2" designation was a remnant from a previous version. This has been corrected.

Line 520: Should "data" be "date"?

Yes, this was a typo. "data" has been changed to "date"

Figure S2. The maize genotypes and N condition should be labeled on the x-axis, as there is a broad range of relative compositions across these samples in the July collection.

The N conditions have been labeled on the x-axis. Since genotype was not included in the statistical analysis we chose not to add these labels.

For the Supplemental Data file, the units of measurement should be provided for the phytohormone and metabolite tabs.

Units have been added to the tabs for each of these measurements in the supplemental data file.
